# C-reactive protein-triglyceride-glucose index versus triglyceride-glucose index in predicting cardiovascular metabolic multimorbidity risk: A cohort study

**DOI:** 10.1371/journal.pone.0340098

**Published:** 2026-02-06

**Authors:** Hongjin Wang, Ming Yang, Wenjing Cai, Hao Zeng, Xin Luo, Zengkai Xu, Jiahuang Wu, Youdong Lin, Zhisheng Wang

**Affiliations:** 1 Department of Cardio-Thoracic Surgery, Longyan First Affiliated Hospital of Fujian Medical University, Longyan, China; 2 First Clinical Medical College, Shandong University of Traditional Chinese Medicine, Jinan, China; 3 Fujian Medical University, Fuzhou, China; 4 Department of Gastroenterology and Anorectal Surgery, Longyan First Affiliated Hospital of Fujian Medical University, Longyan, China; 5 Fuzhou University Affiliated Provincial Hospital, Fuzhou, China; The Chinese University of Hong Kong, HONG KONG

## Abstract

**Background:**

Cardiovascular Metabolic Multimorbidity (CMM), a leading global cause of mortality, lacks evidence on the predictive utility of the novel C-reactive protein-triglyceride-glucose index (CTI), which integrates insulin resistance and inflammation. This study compared CTI with the established Triglyceride-Glucose (TyG) index in predicting CMM risk.

**Methods:**

A cohort of 8,487 adults aged ≥45 from the CHARLS database (2011–2020) was analyzed. Over nine years, CMM events were tracked. Cox regression, restricted cubic spline (RCS), and ROC curve analyses assessed associations between TyG, CTI, and CMM risk. Subgroup analyses evaluated population-specific variations.

**Results:**

Among participants, 1,030 (12.14%) developed CMM. Unadjusted Cox models showed TyG (HR = 1.89, 95%CI 1.73–2.07, *P* < 0.001) and CTI (HR = 1.83, 95%CI 1.70–1.97, *P* < 0.001) predicted CMM; adjusted models confirmed persistence. A dose–response association was observed for both CTI and TyG with CMM risk. In fully adjusted models, the overall dose–response trend remained similar. ROC analysis favored CTI (higher AUC). Subgroup analyses indicated TyG’s association varied by sex, smoking, and hypertension (*P* < 0.05), while CTI’s association differed by age, sex, and hypertension (*P* < 0.05).

**Conclusions:**

Elevated CTI independently correlates with increased CMM risk, demonstrating superior predictive accuracy over TyG. Its linear association highlights potential clinical utility for early CMM risk stratification.

## Introduction

Cardiovascular Metabolic Multimorbidity (CMM) refers to a group of interrelated disease states that include cardiovascular disease (CVD) and metabolic disorders. The coexistence of CVD, diabetes, and stroke is increasing dramatically worldwide [1]. In China, the prevalence of CMM was reported as 11.6%−16.9% [2,3]. The risk of all-cause mortality is 3.7 to 6.9 times higher in individuals with CMM and is associated with a reduced life expectancy of 12–15 years compared to patients without cardiometabolic diseases(CMDs) at age 60 [4]. Patients with CMM face double the risk of death and a significantly lower life expectancy compared to those with single CMDs [5]. The CMM has emerged as a pressing global health challenge. Existing risk models (e.g., Framingham score) have limited predictive efficacy for multimorbidity and require the development of novel biomarkers.

Triglyceride-Glucose (TyG) index, as a reliable marker of insulin resistance (IR), has been shown to predict a single metabolic disease (e.g., diabetes mellitus) or CVD [6–8]. However, the TyG index does not include the inflammatory index, and chronic low-grade inflammation (such as elevated CRP) is the common pathological basis of CMM [9–12]. The C-reactive protein-triglyceride-glucose index (CTI), as a composite index integrating inflammation and metabolism, may more comprehensively reflect CMM risk. CRP, by promoting endothelial dysfunction and atherosclerosis, cooperates with TyG to predict the progression of CMM [13]. However, no study has systematically examined the predictive ability of the CTI for CMM. Both CTI and TyG represent composite metabolic indices that capture dysregulation in lipid–glucose metabolism and are closely associated with insulin resistance. The objective of this study was to compare CTI with the TyG index to determine whether CTI may serve as a potential biomarker for predicting CMM risk and to evaluate its discriminatory performance further.

This study utilized data from the nationally representative China Health and Retirement Longitudinal Study (CHARLS) in a retrospective cohort design. The findings contribute to the development of potential risk indicators for CMM and provide further insight into the contribution of inflammatory and metabolic pathways to its occurrence. Early identification of high-risk individuals and timely implementation of preventive strategies remain essential for reducing CMM burden at both the individual and population levels.

## Materials and methods

### Data source and study population

This cohort study utilized data from the CHARLS, a nationally representative survey targeting middle-aged and older adults (≥ 45 years) in China, launched in 2011. The study employed a biennial follow-up design, with five waves of data collection completed by 2020 [14].

**Fig 1:**
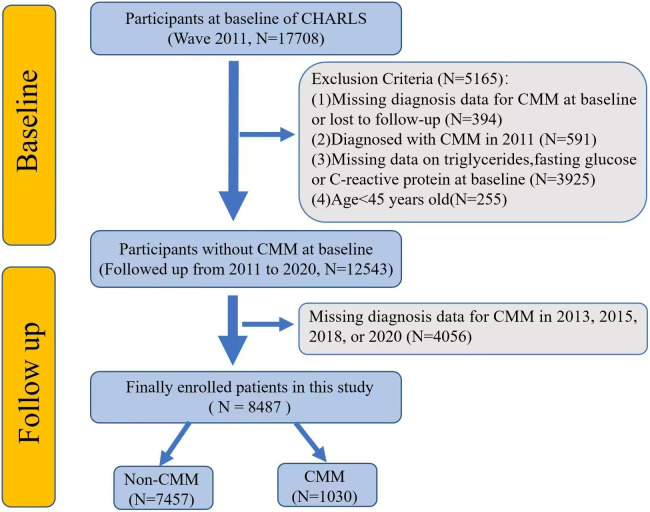
The flowchart of this study. Abbreviation: CHARLS, China Health and Retirement Longitudinal Study. CMM, Cardiovascular Metabolic Multimorbidity.

The study procedure was illustrated in Fig 1. The exclusion criteria were as follows: (1) missing diagnosis data for CMM at baseline or lost to follow-up; (2) diagnosed with CMM in 2011; (3)missing data on triglycerides, fasting glucose or C-reactive protein at baseline; and (4)age < 45 years old. Finally, a total of 8487 participants were included in the study.

The CHARLS project was conducted in accordance with the principles of the Declaration of Helsinki and obtained approval from the Institutional Review Board of Peking University (approval number: IRB00001052–11015). Written informed consent was obtained from all participants prior to enrollment. As this study involved secondary analysis of anonymized CHARLS data, additional ethical approval was not required.

### TyG index and CTI assessment

Biochemical testing was performed at the Youanmen Center for Clinical Laboratory, Capital Medical University. Measurements were obtained from frozen plasma or whole blood using standardized enzymatic colorimetric and immunoturbidimetric assays. The coefficients of variation for triglyceride and glucose assays were below 2%, indicating high analytical precision and reliability [15]. The TyG index was calculated as follows: TyG = ln [TG (mg/dL) × FBG (mg/dL)/ 2] [16]. The formula for calculating the CTI is: CTI = 0.412* Ln (CRP(mg/L)) + TyG [17].

### Assessment of endpoint events

The outcome of this study was the occurrence of CMM, defined as having two or more of the following conditions: heart disease, diabetes, and stroke. The diagnosis of heart disease and stroke was confirmed through self-reported physician-diagnosed questionnaires. Respondents were asked, “Has a doctor ever told you that you had a heart attack, coronary heart disease, angina, congestive heart failure, or other heart problems?” For stroke, the assessment was based on the question, “Has a doctor ever informed you that you had a stroke?” For diabetes, in addition to self-reported diabetes, participants were classified as having diabetes if they met any of the following criteria:(1)Fasting plasma glucose ≥7.0 mmol/L;(2)Random plasma glucose ≥11.1 mmol/L; or (3)HbA1c ≥ 6.5%, based on the American Diabetes Association standards [18]. Participants who developed CMM at any follow-up wave were classified as incident cases and were not followed further for outcome ascertainment. Participants who remained free of CMM continued follow-up until the 2020 survey wave. Because incident CMM was only identified at discrete follow-up waves, the exact onset time was unknown. Therefore, the event time was assigned to the date of the visit at which CMM was first detected. Participants without CMM were censored at their last visit.

### Data collection

The data included in this study were age, gender, body mass index (BMI), smoking, drinking, marital status, residential area, education, hypertension, and cancer. Smoking and drinking were divided into three categories: never, ever and current. Specifically, current smokers/drinkers were defined as those who currently smoke tobacco or drink alcohol at the time of the baseline survey. Former smokers/drinkers were defined as those who reported having quit smoking or drinking at the time of the baseline survey. This category did not distinguish the duration or reason for cessation. Never smokers/drinkers were those who reported never having smoked or drunk alcohol. Marital status was divided into two categories: others and married. The residential area was classified into urban and rural, while education level was divided into four categories: below primary school, primary school, middle school, and high school and above. Hypertension and cancer were defined based on a self-reported history provided by the participants.

### Statistical analysis

The Shapiro-Wilk test was utilized to assess the normality of the data. In this study, the continuous variables were found to be normally distributed and therefore expressed as Mean ± SD, while categorical data were expressed as n (%). Comparisons for categorical variables were made using either the Chi-square test or Fisher’s exact test. Continuous variables were analyzed by using an independent sample t-test.

To examine the relationship between the TyG index, CTI, and CMM risk, we established both univariate and multivariate Cox regression models. The TyG index and CTI were incorporated into the models as continuous and categorical variables, respectively. The quartile ranges for the TyG index were as follows: Q1: 6.625–8.234, Q2: 8.235–8.593, Q3: 8.594–9.028, and Q4: ≥ 9.029. The quartile ranges for CTI were Q1: 7.120–8.144, Q2: 8.145–8.645, Q3: 8.646–9.203, and Q4: ≥ 9.204. Model 1 was unadjusted; Model 2 was adjusted for age and gender; and Model 3 was adjusted for Model 2 plus education, marital status, BMI, smoking, drinking, cancer, residential area, and hypertension.

To facilitate a more intuitive analysis of the cumulative risk of CMM associated with the TyG index and CTI over time, Kaplan–Meier survival curves were employed. We divided the TyG index and CTI into categorical variables according to the quartile ranges to facilitate a better comparison of CMM incidence differences among the groups. In constructing the model, we defined the time axis at two, four, seven, and nine years within the observation period. The vertical axis represented the cumulative risk of CMM.

To provide a more intuitive demonstration of the relationships among TyG index, CTI, and CMM occurrences, we established Restricted Cubic Spline(RCS) models based on the Cox regression models, following the Bayesian Information Criterion for model selection. The Likelihood Ratio Test was used to evaluate the non-linear relationships in RCS models. The adjustment factors for these three RCS models were consistent with the adjustment factors in the three Cox regression models.

To compare the predictive efficacy of the TyG index and CTI in assessing CMM risk, we conducted a Receiver Operating Characteristic (ROC) analysis. We constructed ROC curves at two-, four-, seven-, and nine-year intervals to evaluate predictive performance at different time points and applied DeLong’s test to compare the areas under the curves(AUC) for the TyG index and CTI.

To further evaluate the predictive ability of TyG index and CTI for CMM risk across different populations, we conducted a subgroup analysis. The variables included age, gender, smoking status, drinking status, BMI, and hypertension. Furthermore, we conducted a sensitivity analysis by excluding patients diagnosed with heart disease, stroke, or diabetes in 2011 to enhance the robustness of our findings. All covariates, including those used in the Cox regression models and subgroup analyses were defined and collected at baseline (the 2011 survey wave). Additionally, because CHARLS provides survey weights and design variables, we evaluated their applicability; however, the present analysis did not use sampling weights as our objective was etiological association estimation rather than population-level prevalence inference.

This study employed the R (4.3.0) and MedCalc(20.215) statistical analysis software. All tests were two-sided, and a significance level of *P* < 0.05 was considered statistically significant.

## Results

### Participant characteristics

Table 1 presents the overall and stratified characteristics of subjects based on the incidence of CMM events after a nine-year follow-up period. The mean age of the study cohort was 58.08 years, with women comprising 54.60% of the subjects. A total of 1030 (12.14%) of the 8487 subjects developed CMM. In this cohort study, the total person-years of follow-up amounted to 73,557.75, with an average follow-up duration of 8.67 years. Table 1 indicates that patients in the CMM group exhibited the following characteristics: older age, a higher proportion of women, increased BMI, a history of never or ever smoking and drinking, single marital status, urban living, elevated blood pressure, and elevated C-reactive protein. Furthermore, the TyG index was significantly higher in the CMM group at 8.92 ± 0.63 compared to 8.64 ± 0.60 in the non-CMM group (*P* < 0.001). The CTI index also showed a significant increase in the CMM group, with values of 9.08 ± 0.78 compared to 8.67 ± 0.76 in the non-CMM group (*P* < 0.001). No significant difference was observed between the two groups regarding education level and comorbid tumors (all *P* > 0.05).

**Table 1:** Basic characteristics of the population included in this study.

Characteristics	Total	Non-CMM	CMM	*P*-value
**No. of participants**	8487	7457	1030	
**Age, mean ± SD, years**	58.08 ± 8.58	57.76 ± 8.57	60.38 ± 8.34	<0.001
**Gender, n(%)**				0.005
Female	4634 (54.60%)	4030 (54.04%)	604 (58.64%)	
Male	3853 (45.40%)	3427 (45.96%)	426 (41.36%)	
**BMI, mean ± SD, kg/m2**	24.17 ± 3.08	23.95 ± 3.21	25.73 ± 1.88	<0.001
**Smoking, n(%)**				<0.001
Never	5307 (62.53%)	4628 (62.06%)	679 (65.92%)	
Ever	669 (7.88%)	556 (7.46%)	113 (10.97%)	
Current	2511 (29.59%)	2273 (30.48%)	238 (23.11%)	
**Drink, n(%)**				<0.001
Never	5230 (61.62%)	4582 (61.45%)	648 (62.91%)	
Ever	641 (7.55%)	535 (7.17%)	106 (10.29%)	
Current	2616 (30.82%)	2340 (31.38%)	276 (26.80%)	
**Marital status, n (%)**				0.001
Others	855 (10.07%)	722 (9.68%)	133 (12.91%)	
Married	7632 (89.93%)	6735 (90.32%)	897 (87.09%)	
**Residential area, n (%)**				0.005
Urban	2886 (34.00%)	2496 (33.47%)	390 (37.86%)	
Rural	5601 (66.00%)	4961 (66.53%)	640 (62.14%)	
**Education, n (%)**				0.204
Below Primary School	3959 (46.65%)	3466 (46.48%)	493 (47.86%)	
Primary School	1833 (21.60%)	1596 (21.40%)	237 (23.01%)	
Meddle School	1780 (20.97%)	1588 (21.30%)	192 (18.64%)	
High school and above	915 (10.78%)	807 (10.82%)	108 (10.49%)	
**Hypertension, n (%)**	3688 (43.45%)	3015 (40.43%)	673 (65.34%)	<0.001
**Cancer, n (%)**	70 (0.82%)	59 (0.79%)	11 (1.07%)	0.357
**CTI index, mean ± SD**	8.72 ± 0.77	8.67 ± 0.76	9.08 ± 0.78	<0.001
**TyG index, mean ± SD**	8.67 ± 0.61	8.64 ± 0.60	8.92 ± 0.63	<0.001
**CRP, mean ± SD, mg/L**	2.35 ± 5.91	2.25 ± 5.69	3.09 ± 7.26	<0.001

Abbreviation: CMM: Cardiovascular Metabolic Multimorbidity; BMI: Body Mass Index; CTI index: C-reactive protein-Triglyceride-glucose index; TyG index: Triglyceride-Glucose index; CRP: C-Reactive Protein.

### The relationship between TyG index, CTI, and CMM

For the TyG index, each additional unit is associated with an 89% increase in CMM risk (Model 1: hazard ratio (HR) = 1.89, 95% CI 1.73–2.07, p < 0.001). Models 2 (HR = 1.92, 95% CI 1.75–2.10, *P* < 0.001) and Model 3 (HR = 1.76, 95% CI 1.60–1.93, *P* < 0.001) also demonstrate a significant association between elevated TyG and increased CMM risk. Similarly, for CTI, each additional unit corresponds to an 83% increase in CMM risk (Model 1: HR = 1.83, 95% CI 1.70–1.97, *P* < 0.001). Model 2 (HR = 1.82, 95% CI 1.69–1.96, *P* < 0.001) and Model 3 (HR = 1.69, 95% CI 1.56–1.82, *P* < 0.001) further affirm the association between elevated CTI and increased CMM risk. The quartiles of the TyG index (Q2, Q3, and Q4) indicate a gradually increasing trend in CMM risk across all three models. For CTI, in all three models, Q2, Q3, and Q4 exhibited significant associations with a rising risk of CMM (Table 2).

**Table 2:** Cox regression models for the association between the TyG index, the CTI and cardiovascular metabolic multimorbidity risk.

Categories	Model 1			Model 2			Model 3		
	HR	95% Cl	*P*	HR	95% Cl	*P*	HR	95% Cl	*P*
**TyG index**									
Continuous	1.89	1.73 - 2.07	<0.001	1.92	1.75 - 2.10	<0.001	1.76	1.60 - 1.93	<0.001
Quartile									
Q1	Reference			Reference			Reference		
Q2	1.55	1.25 - 1.92	<0.001	1.54	1.24 - 1.91	<0.001	1.46	1.17 - 1.81	<0.001
Q3	2.17	1.77 - 2.67	<0.001	2.13	1.73 - 2.61	<0.001	1.88	1.53 - 2.32	<0.001
Q4	3.19	2.62 - 3.87	<0.001	3.19	2.63 - 3.88	<0.001	2.71	2.22 - 3.30	<0.001
**CTI**									
Continuous	1.83	1.70 -1.97	<0.001	1.82	1.69 - 1.96	<0.001	1.69	1.56 - 1.82	<0.001
Quartile									
Q1	Reference			Reference			Reference		
Q2	1.74	1.39 - 2.19	<0.001	1.68	1.34 - 2.11	<0.001	1.59	1.27 - 2.00	<0.001
Q3	2.36	1.90 - 2.93	<0.001	2.24	1.81 - 2.79	<0.001	1.98	1.59 - 2.46	<0.001
Q4	4.11	3.35 - 5.03	<0.001	3.95	3.22 - 4.84	<0.001	3.32	2.70 - 4.08	<0.001

Model 1: unadjusted.

Model 2: adjusted for age and gender.

Model 3: adjusted for Model 2 plus education, marital status, body mass index, smoking, drinking, cancer, residential area, and hypertension.

### Cumulative risk of CMM: analysis using Kaplan-Meier survival curves

**Fig 2:**
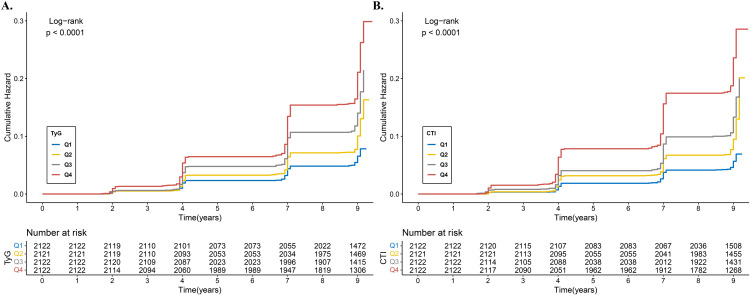
Kaplan–Meier survival curves for cumulative cardiovascular metabolic multimorbidity risk by TyG (A) and CTI (B). Abbreviation: TyG, triglyceride–glucose index; CTI, C-reactive protein-triglyceride-glucose index.

Fig 2A demonstrated that the cumulative CMM risk increased with higher TyG quartiles, a trend that was statistically significant (Log-rank test: Chi-square = 178.4675, *P* < 0.001). Likewise, Fig 2B revealed that the cumulative CMM risk also rose with higher CTI quartiles, which was also statistically significant (Log-rank test: Chi-square = 263.2313, *P* < 0.001).

### Analysis of relationships between TyG, CTI, and CMM risk using RCS models

**Fig 3:**
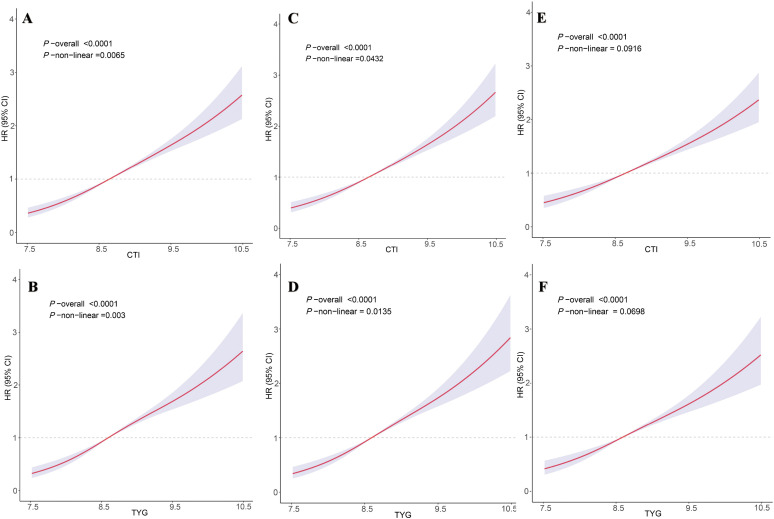
Restricted cubic spline models analyzed the relationship between TyG (A)(C)(E) and CTI (B)(D)(F) and cardiovascular metabolic multimorbidity risk. (A)(B) represents unadjusted covariates; (B)(D) are adjusted for age and gender; and (E)(F) are adjusted for age, gender, education, marital status, BMI, smoking, drinking, cancer, residential area, and hypertension. The solid line and purple area represent estimates and their corresponding 95% confidence intervals (CIs), respectively.

Fig 3A shows that the TyG index demonstrated an overall positive dose–response association with CMM risk, with modest non-linearity supported by the LR test (overall *P* < 0.0001; non-linear *P* = 0.003). After adjustment, a similar increasing dose–response pattern was observed (Fig 3C), and the curve remained broadly upward with only small deviations (Fig 3E). Fig 3B illustrates a comparable positive dose–response association for CTI (overall *P* < 0.0001; non-linear *P* = 0.0065), which persisted after adjustment (Fig 3D and Fig 3F), with non-linearity detected but without meaningful changes to the overall upward trend.

### Comparison of predictive efficacy of TyG index and CTI for CMM risk events at various time points

**Fig 4:**
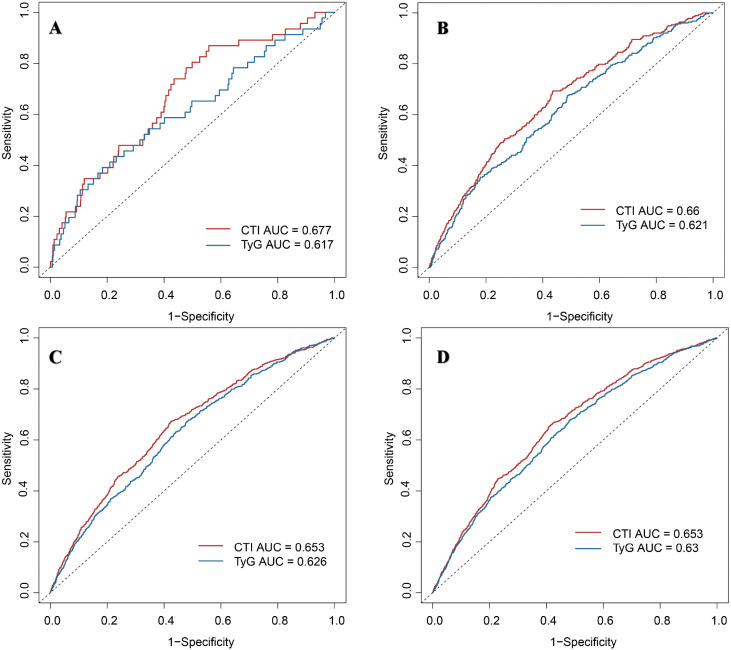
Receiver operating characteristic curves compared the predictive efficacy of the triglyceride–glucose index and c-reactive protein-triglyceride-glucose index for cardiovascular metabolic multimorbidity risk events at two- (A), four- (B), seven- (C), and nine-year (D).

At 2 years (Fig 4A), the AUC for TyG was 0.64 (95% CI: 0.49–0.78), compared with 0.65 (95% CI: 0.52–0.78) for CTI, yielding an absolute AUC difference of 0.01 (*P* < 0.05). At 4 years (Fig 4B), AUCs were 0.62 (95% CI: 0.58–0.67) for TyG and 0.66 (95% CI: 0.62–0.71) for CTI, with an absolute difference of 0.04 (*P* < 0.05). At 7 years (Fig 4C), AUCs were 0.62 (95% CI: 0.59–0.64) and 0.65 (95% CI: 0.63–0.68) for TyG and CTI, respectively, resulting in an absolute difference of 0.03 (*P* < 0.05). At 9 years (Fig 4D), AUCs remained modest—0.64 (95% CI: 0.61–0.66) for TyG and 0.66 (95% CI: 0.64–0.69) for CTI—with an absolute difference of 0.02 (*P* < 0.05).

### Subgroup analysis of TyG index and CTI in predicting CMM risk

**Fig 5:**
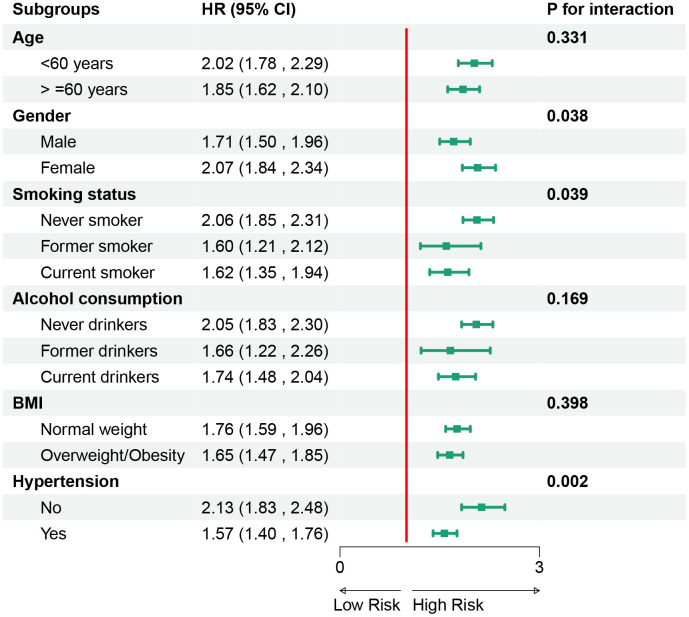
Subgroup analysis of TyG in predicting cardiovascular metabolic multimorbidity risk. Abbreviation: TyG, triglyceride–glucose index; BMI, body mass index.

**Fig 6:**
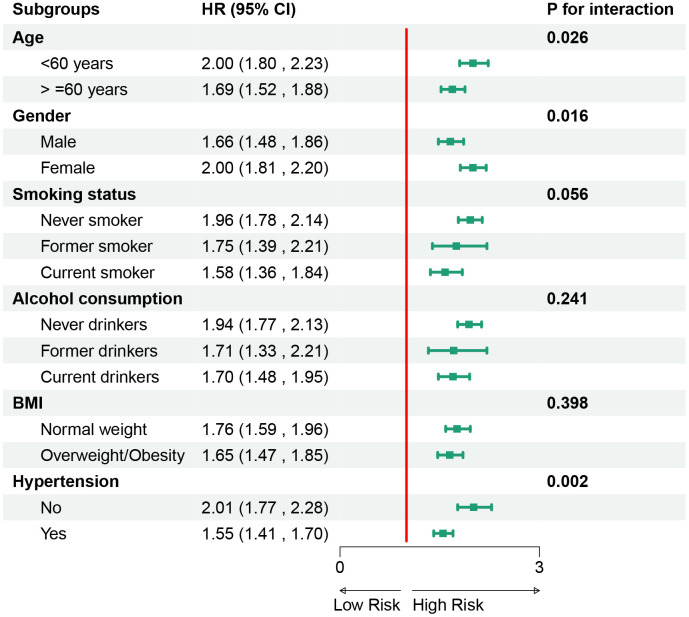
Subgroup analysis of CTI in predicting cardiovascular metabolic multimorbidity risk. Abbreviation: CTI, c-reactive protein-triglyceride-glucose index; BMI, body mass index.

Fig 5 illustrates the subgroup analyses of TyG in predicting the risk of CMM. It was observed that TyG and CMM risk were not influenced by age, alcohol consumption, or BMI (p > 0.05 for interaction), but were affected by gender, smoking status, and hypertension(p < 0.05 for interaction). Similarly, Fig 6 presents the subgroup analyses for CTI. It was found that CTI and CMM risk were also not significantly influenced by factors such as smoking status, alcohol consumption, and BMI (p > 0.05 for interaction), but were affected by age, gender, and hypertension(*P* < 0.05 for interaction).

### Sensitivity analysis

We performed a sensitivity analysis restricted to participants free of all three CMM component diseases (heart disease, diabetes, and stroke) at baseline (n = 6,540). In this sub-cohort, the associations between both the TyG index (HR = 1.59, 95% CI 1.34–1.89) and CTI (HR = 1.59, 95% CI 1.39–1.81) with incident CMM remained significant and of similar magnitude to the primary analysis, reinforcing the robustness of our findings (S1 Table).

## Discussion

In this study, we evaluated the prognostic utility of CTI for CMM risk and compared its performance with the TyG index. The results demonstrated that both indices remained significantly linked to elevated CMM risk after comprehensive adjustment for multiple confounders. Notably, despite having a lower HR, CTI showed superior predictive ability for CMM risk compared to the TyG index.

Previous studies have demonstrated that a high TyG index correlates with an increased risk of CMM, aligning with our findings. One study analyzing 7,970 participants found that a high TyG index was associated with a higher incidence risk of CMM [19]. A study analyzing 6,451 participants found that an elevated TyG index was associated with an increased risk of CMM. Additionally, indicators derived from the TyG index, such as the TyG body mass index (TyG-BMI) and TyG-waist circumference (TyG-WC), were also linked to CMM risk [20]. Our study indicates that the TyG index is influenced by gender, smoking status, and hypertension. In conclusion, many existing studies affirm the positive correlation between the TyG index and CMM risk.

This study differs from previous studies, which are the first to determine an association between CTI and CMM risk, and also includes a comparative analysis with the TyG index. In the Cox regression analysis, we found that CTI showed a lower HR than TyG index. The reason for this result may be that insulin resistance (TyG core) is the core driving factor of CMM [21–25], and its sole effect may be manifested as a stronger risk gradient (high HR).In the middle-aged Chinese population, hs-CRP was associated with an increased risk of developing CMM [26]. Annapurna et al. [27] found no significant association between serum CRP levels and other cardiovascular diseases (myocardial infarction, coronary heart disease, heart failure, atherosclerosis).CRP is a sensitive marker of inflammatory processes and the severity of atherosclerosis in cardiovascular and cerebrovascular diseases, but its elevation may be a result of disease status rather than a cause [28]. These suggest that inflammation (CRP) may act as the “second hit, “ heightening risk on top of metabolic abnormalities, but only contributing small individual effects, thereby diluting the unit HR when integrated. In conclusion, chronic low-grade inflammation serves primarily as an “amplifier” rather than a starting factor for metabolic damage, which requires further exploration in future studies. Additionally, future research can investigate various higher-quality composite indices to predict CMM risk.

Currently, the mechanism by which the TyG index and CTI predict CMM risk is unclear. This may relate to glucose metabolism, lipid metabolism, the inflammatory response, oxidative stress, and other factors. Firstly, it is important to note that as a composite index integrating triglycerides and blood glucose, both can reflect the core metabolic abnormalities associated with CMM development [29,30]. Secondly, the elevated levels of the TyG index and CTI may indicate the body’s IR status. Studies have shown that the TyG index can effectively predict the risk of IR and cardiac metabolic risk factors [31].In one study, the TyG index was shown to be a reliable surrogate marker of insulin resistance (IR) and had a significant association with abnormal fat accumulation and markers of atherosclerosis [32]. The CTI further integrates the metabolic-inflammatory interplay by introducing CRP, which is not only an inflammatory marker but also promotes CMM progression through its direct involvement in vascular injury, metabolic disorders, and thrombosis [33]. In summary, the TyG index and CTI, as composite indicators of blood, reflect IR and inflammatory status. The mechanism by which they predict CMM risk remains to be explored further, possibly involving various metabolic and oxidative stress-related processes.

The TyG index, as a well-validated surrogate of insulin resistance, offers high simplicity and reproducibility, focusing squarely on core metabolic dysfunction. Its primary limitation in the context of CMM is its inability to reflect the contributory role of chronic inflammation. The CTI, by design, addresses this limitation by combining metabolic and inflammatory information, providing a more holistic risk assessment. This integration is its key strength, likely explaining its superior discriminative power. However, this comes with potential trade-offs. The inclusion of CRP, a non-specific acute-phase reactant that can be transiently elevated by conditions unrelated to cardiometabolic disease (e.g., minor infections), may introduce noise and reduce the specificity of the CTI. Furthermore, the calculation of CTI is slightly more complex than TyG. The choice between them may depend on the clinical or research context: TyG remains a powerful and straightforward metabolic marker, while CTI should be considered when a more comprehensive assessment integrating inflammation is desired.

As the first study to explore the association between CTI and CMM, a new idea for disease risk assessment was opened through comparative analysis with the TyG index. The innovative multi-model collaborative analysis strategy was incorporated into the methodology, utilizing survival analysis, nonlinear fitting, and predictive performance evaluation to enhance the scientific rigor and persuasiveness of the research results significantly. Based on data from large national cohorts, the study design effectively avoids sample selection bias and ensures that the conclusions are representative of the population. From the perspective of application value, the research results not only provide a reliable risk assessment tool for clinical practice but also offer data support for public health decision-making, which is highly significant for promoting hierarchical diagnosis and treatment as well as precise prevention.

This study has several limitations. First, the variables used in our subgroup analyses were measured at a single time point (baseline). While this is standard practice in cohort studies for establishing risk prediction models, it does not account for potential changes in these risk factors (e.g., a participant developing hypertension or quitting smoking) during the follow-up period. Such changes could influence the long-term generalizability of our risk estimates. Second, detailed information on long-term medication use was limited in CHARLS, preventing full adjustment for treatments such as antihypertensive, antidiabetic, or lipid-lowering drugs. As these medications may affect CRP and metabolic markers, residual confounding cannot be excluded and the associations of CTI and TyG with CMM risk should be interpreted with caution. Third, Survey weights were not applied, which may limit population-level generalizability. Fourth, because the onset was interval censored and assigned to the detection visit, some misclassification of event timing is possible, which may introduce bias and slightly affect hazard estimates. Finally, other potential confounders such as diet, physical activity, socioeconomic factors, and genetic predispositions were not available, so residual confounding may remain. Given these limitations and remaining methodological issues, CTI should be interpreted as an emerging indicator rather than a clinically established tool.

## Conclusion

The CTI significantly predicted CMM risk and modestly outperformed the TyG index. While a promising research tool for risk assessment, its clinical utility needs external validation and calibration before clinical use.

## Supporting information

S1 Table Supplemental table. (DOCX)
