# Supplementary material for: C-reactive protein-triglyceride-glucose index versus triglyceride-glucose index in predicting cardiovascular metabolic multimorbidity risk: A cohort study
Source: PLoS One. 2026 Feb 6;21(2):e0340098. doi: 10.1371/journal.pone.0340098 (PMC12880693; doi:10.1371/journal.pone.0340098)
Supplement: S1 Table — (DOCX) [file pone.0340098.s001.docx]

**Table S1.** Sensitivity analysis restricted to participants free of baseline diabetes, heart disease, and stroke: associations of TyG and CTI with incident CMM.

| **Categories** | **Model 1** | | |  | **Model 2** | | |  | **Model 3** | | |  |
| --- | --- | --- | --- | --- | --- | --- | --- | --- | --- | --- | --- | --- |
|  | **HR** | **95% Cl** | ***P*** | | **HR** | **95% Cl** | ***P*** | | **HR** | **95% Cl** | ***P*** | |
| **TyG index** |  |  |  | |  |  |  | |  |  |  | |
| Continuous | 1.79 | 1.53 - 2.10 | <0.001 | | 1.83 | 1.56 - 2.14 | <0.001 | | 1.59 | 1.34 - 1.89 | <0.001 | |
| Quartile |  |  |  | |  |  |  | |  |  |  | |
| Q1 | **Reference** |  |  | | **Reference** |  |  | | **Reference** |  |  | |
| Q2 | 1.33 | 0.97 - 1.84 | 0.08 | | 1.34 | 0.97 - 1.85 | 0.07 | | 1.30 | 0.93 – 1.82 | 0.12 | |
| Q3 | 1.95 | 1.44 - 2.63 | <0.001 | | 1.97 | 1.45 - 2.66 | <0.001 | | 1.79 | 1.30 - 2.45 | <0.001 | |
| Q4 | 2.41 | 1.80 - 3.22 | <0.001 | | 2.47 | 1.85 - 3.31 | <0.001 | | 2.04 | 1.49 – 2.78 | <0.001 | |
| **CTI** |  |  |  | |  |  |  | |  |  |  | |
| Continuous | 1.77 | 1.56 -2.00 | <0.001 | | 1.75 | 1.55 - 1.99 | <0.001 | | 1.59 | 1.39 - 1.81 | <0.001 | |
| Quartile |  |  |  | |  |  |  | |  |  |  | |
| Q1 | **Reference** |  |  | | **Reference** |  |  | | **Reference** |  |  | |
| Q2 | 2.08 | 1.47 - 2.95 | <0.001 | | 2.03 | 1.43 – 2.87 | <0.001 | | 2.03 | 1.40 - 2.93 | <0.001 | |
| Q3 | 2.86 | 2.05 – 3.99 | <0.001 | | 2.75 | 1.97 – 3.85 | <0.001 | | 2.56 | 1.80 – 3.64 | <0.001 | |
| Q4 | 3.49 | 2.52 – 4.83 | <0.001 | | 3.40 | 2.45 - 4.71 | <0.001 | | 2.93 | 2.07 - 4.16 | <0.001 | |

Model 1: unadjusted.

Model 2: adjusted for age and gender.

Model 3: adjusted for Model 2 plus education, marital status, body mass index, smoking, drinking, cancer, residential area, and hypertension.

Abbreviation: TyG: Triglyceride-Glucose; CTI: C-reactive protein-Triglyceride-glucose Index; HR: Hazard Ratio; CI: Confidence Interval.
